# Electrochemical Detection of Cortisol by Silver Nanoparticle-Modified Molecularly Imprinted Polymer-Coated Pencil Graphite Electrodes

**DOI:** 10.1021/acsomega.3c02472

**Published:** 2023-08-02

**Authors:** Nemah Abu Shama, Süleyman Aşır, Ilgım Göktürk, Fatma Yılmaz, Deniz Türkmen, Adil Denizli

**Affiliations:** † Department of Life Sciences, Faculty of Natural Sciences, Ben-Gurion University, Beer Sheva 84105, Israel; ‡ Department of Materials Science and Nanotechnology Engineering, Near East University, Mersin 10 Turkey, Nicosia 99138, North Cyprus; § Department of Chemistry, Hacettepe University Ankara, 06800, Turkey; ∥ Department of Chemistry and Chemical Processing Technologies, Bolu Abant Izzet Baysal University, Bolu 14030, Turkey

## Abstract

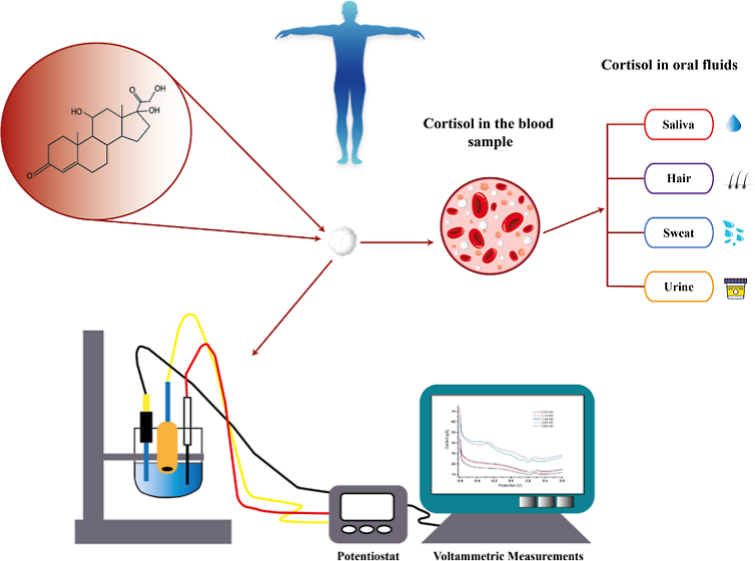

The sensitive cortisol detection by an electrochemical sensor based on silver nanoparticle-doped molecularly imprinted polymer was successfully improved. This study describes the method development for cortisol detection in both aqueous solution and biological samples using molecularly imprinted poly(hydroxyethyl methacrylate-*N*-methacryloyl-(l)-histidine methyl ester)-coated pencil graphite electrodes modified with silver nanoparticles (AgNPs) by differential pulse voltammetry (DPV). The cortisol-imprinted pencil graphite electrode (PGE) has a large surface area because of doped AgNPs with enhanced electroactivity. The prepared molecularly imprinted polymer was characterized by scanning electron microscopy. The DPV response of the synthesized electrode with outstanding electrical conductivity was clarified. Cortisol-imprinted polymer-coated PGEs (MIP), cortisol-imprinted polymer-coated PGEs with AgNPs (MIP@AgNPs), and nonimprinted polymer-coated PGEs with AgNPs (NIP@AgNPs) were evaluated for sensitive and selective detection of cortisol in aqueous solution. Five different cortisol concentrations (0.395, 0.791, 1.32, 2.64, and 3.96 nM) were applied to the MIP@AgNPs, and signal responses were detected by the DPV with a regression coefficient (*R*^2^) value of 0.9951. The modified electrode showed good electrocatalytic activity toward cortisol for the linear concentration range from 0.395 to 3.96 nM, and a low limit of detection was recorded as 0.214 nM. The results indicate that the MIP@AgNPs sensor has great potential for sensitive and selective cortisol determination in biological samples.

## 1 Introduction

**Figure 1:**
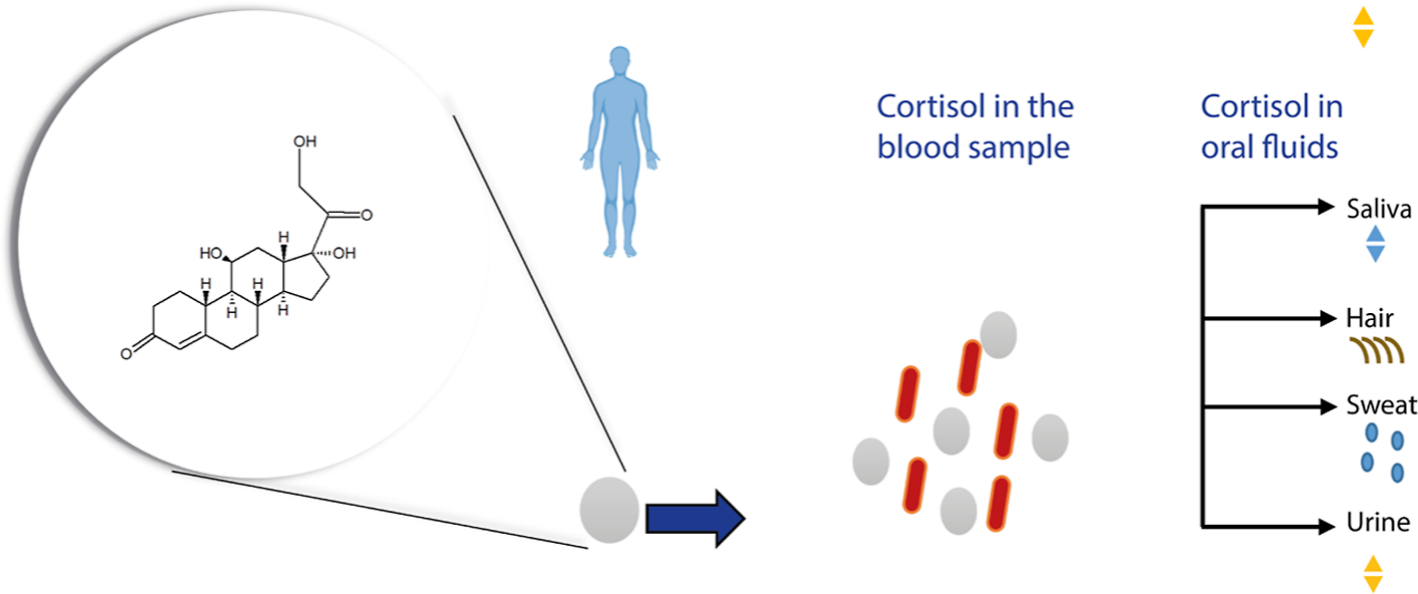
Presence of free cortisol released into the blood and then distributed to the oral fluids by diffusion.

Human life and behavior have the most attention in all different research disciplines. Cortisol indicates the life-satisfying adrenal hormone, which is vital to homeostasis preservation. Cortisol is a steroid hormone; its chemical structure comprises a four-ringed nucleus: three cyclohexane rings, and one cyclopentane ring with five carbon atoms, and the functional groups are hydroxyl, keto, and 17-beta-hydroxy groups, as shown in Figure 1.^1^ The hypothalamic adrenal organism produces it, which is manufactured as a part of the human body’s tension response. That is why cortisol, also called a stress hormone, affects and controls several physical and biological activities, for instance, glucose level, heart reductions, the activation of the central nervous system, immune system responses, and blood pressure.^2^ It also plays a role in the sleep–wake cycle and influences memory and learning. Accurately detecting cortisol levels is crucial for medical diagnosis and monitoring as abnormal cortisol levels can indicate underlying health conditions.^3^ Elevated cortisol levels are associated with conditions such as Cushing’s syndrome, chronic stress, and certain tumors, while low cortisol levels can be indicative of Addison’s disease or adrenal insufficiency.^4^ Monitoring cortisol levels is essential in diagnosing and managing these conditions and evaluating the efficacy of treatments, such as hormone replacement therapy. However, traditional methods for cortisol detection, such as UV spectroscopy,^5^ immunoassays or chromatographic techniques,^6^ and capillary electrophoresis,^7^ have certain limitations. These methods often require sophisticated equipment, time-consuming sample preparation, and trained personnel, making them less suitable for rapid, on-site analysis. Additionally, they may have sensitivity, selectivity, and cost-effectiveness limitations. Electrochemical methods have emerged as promising alternatives for cortisol detection due to their inherent advantages.^8^ These methods offer high sensitivity and selectivity, allowing for accurate cortisol detection even at low concentrations. They also demonstrate fast response times, enabling real-time or near-real-time monitoring of cortisol levels. Moreover, electrochemical sensors can be miniaturized, making them suitable for portable and point-of-care applications.^9^ Sensors can be cost-effective and compatible with complex biological samples, facilitating their integration into wearable devices or personalized healthcare systems. By addressing the limitations of conventional techniques, electrochemical methods provide a valuable tool for sensitive and rapid cortisol detection, enabling improved medical diagnosis, treatment monitoring, and personalized healthcare approaches. Hence, electrochemical electrode sensors are one of the most popular methods for detecting cortisol; they demonstrate a remarkable capacity for high sensitivity, enabling the precise detection of very low concentrations of the targeted analyte, particularly when it exists in trace quantities.^10^ Moreover, through meticulous design, these sensors can achieve an elevated level of selectivity, effectively mitigating the influence of interfering substances and safeguarding the accuracy of the measurement. Additionally, electrochemical sensors boast commendable attributes such as rapid response times, cost-effectiveness, and compatibility with complex sample matrices.^11,12^ A recent study showed that the application of gold nanoparticles and magnetic functionalized reduced graphene oxide (AuNPs/MrGO) based on an immunosensor improved the detection of cortisol. The immunosensor showed an excellent analytical performance range of 0.1–1000 ng/mL with a limit of detection (LOD) of 0.05 ng/mL.^13^ Another research study worked on the usage of molecularly imprinted polymers (MIPs) in the molecule detection and biomolecules by forming antibodies as receptors and, because of the MIPs’ high disabilities in surroundings and a long electrode shelf life, proved their capacity to detect cortisol molecules even at very low concentrations.^14^ MIPs offer mechanical and thermal stability even at extreme pH and temperature values, are simple to prepare/design, reusable, and durable, can be stored/transported at ambient temperatures, and have a longer shelf-life.^15^ As a result, MIPs have been widely used in the design of biosensors.^16−20^ In recent years, strategies based on nanomaterials to produce electrochemical sensors such as gold and carbon nanoparticles, which give fast and real-time analyses of cortisol levels in many biological samples, for instance, urine, plasma, and saliva, were improved.^21^ Therefore, electrochemical detection is a reliable method for quantifying cortisol in biological matrices with the benefit of a quick response.^22^ Other studies describe electrochemical techniques including amperometry, electrochemical impedance spectroscopy (EIS), square wave voltammetry (SWV), and cyclic voltammetry (CV) for measuring cortisol levels in tear fluid. They discovered that label-free EIS was the most accurate way to measure the amount of cortisol in tear fluid with a 10% relative standard deviation and a lower LOD of 59.76 nM.^23^ In general, electrochemical technology substitutes the optical techniques in recent research, especially in detecting biomolecules. The principle of voltammetric electrochemical measurement is based on the relationship between the electrical properties of an electroactive species and its concentration in a solution. An electroactive species can undergo oxidation or reduction reactions at the electrode surface when it is added to an electrochemical cell that includes an electrode and an electrolyte. These reactions produce an electrical current. The current generated can be monitored and used to calculate the concentration of the electroactive species in the solution by adjusting the electrode’s applied voltage. CV and differential pulse voltammetry (DPV) are the most familiar and sensitive voltammetric measurements. This research focused on the attempt to reach a highly sensitive and selective cortisol detection using cortisol-imprinted poly(hydroxyethyl methacrylate-*N*-methacryloyl-(l)-histidine methyl ester) poly(HEMA-MAH)-coated pencil graphite electrodes (PGEs) modified with silver nanoparticles by DPV. In this technique, the significant step is the determination of cortisol at ultralow concentrations in order to achieve a low LOD. Three types of electrodes were tested for sensitivity and selectivity studies: cortisol-imprinted polymer-coated PGEs (MIP), cortisol-imprinted polymer-coated PGEs with AgNPs (MIP@AgNPs), and nonimprinted polymer-coated PGEs with AgNPs (NIP@AgNPs).

Several reports have examined the use of modified MIP electrode sensors.^24−27^ This study applied a polymer-containing *N*-methacryloyl-(l)-histidine methyl ester (MAH) to create a PGE surface that could initiate electron transfer and enhance sensitivity for cortisol detection. The addition of AgNPs was also evaluated to improve sensitivity in the MIP@AgNPs system further. The selectivity efficiency of the system was determined by measuring cortisol detection in the presence of competitors such as fluticasone and clobetasol. The use of MIPs has the potential to achieve higher sensitivity and selectivity compared to traditional methods. Additionally, using AgNPs on the electrodes can enhance electrocatalytic activity and reduce the detection limit. This research presents a new approach to cortisol detection that could be applied in fields such as clinical diagnostics and environmental monitoring. Furthermore, the system was able to differentiate cortisol in complex matrix samples such as human blood plasma, demonstrating its potential for real sample detection.

## 2 Experimental Section

### 2.1 Materials and Reagents

Silver nitrate, sodium citrate, l-histidine methyl ester, and methacryloyl chloride were obtained from Merck (Darmstadt, Germany). Ethylene glycol dimethacrylate (EDMA), 2-hydroxyethyl methacrylate (HEMA), and azoisobisbutyronitrile (AIBN) were purchased from Sigma (Chemical Co., ABD). Cortisol (98%), fluticasone, clobetasol propionate, methanol, and acetic acid were supplied from Merck (Darmstadt, Germany). All of the aqueous solutions were created using deionized (DI) water (18.2 MΩ cm) and pure lab Ultra Analytic (ELGA Lab Water, UK). The pH was measured using a pH meter, and solution preparations were done in an ultrasonic water bath. Pencil tips (Tombow 2.0, HB), purchased from nearby stationery stores, were used as pencil graphite electrodes (PGEs).

### 2.2 Apparatus

DPV measurements were performed using an AUTO LAB PGSTAT101 potentiostat/galvanostat with NOVA 2.1.2 software (Pine Instrument Company, the Netherlands). Electrochemical measurements were performed using an electrochemical cell comprising three main electrodes. A platinum wire served as the counter electrode (CE), which is also known as the auxiliary electrode. The reference electrode (RE), consisting of Ag/AgCl, may be seen on the second electrode (3 M KCl). The working electrode (WE) is the third electrode. In order to prevent any potential oxidation reactions of the analyte, the solutions were prepared freshly before each analysis for each concentration of cortisol. Additionally, all detection techniques were also tested at 25 °C. DPV measurements were recorded with a potential of −0.6 V, a stop potential of +0.6 V, a pulse time of 0.5 s, a step height of 0.005 mV, and an amplitude of 0.025 V.

### 2.3 Stock Solutions and Real Sample Preparations

The buffer solution was formulated using potassium phosphate and potassium dihydrogen phosphate to achieve a pH level of 7.4, which is within the physiological range. Next, the cortisol standard stock solution was prepared by dissolving the appropriate amount of cortisol in a suitable volume of phosphate buffer solution (PBS). The same procedure was followed to prepare solutions of fluticasone and clobetasol propionate by dissolving the respective amounts in PBS.

The collection process of human blood samples was performed on volunteers in the NEU Hospital. For this purpose, the approval of the Institute Ethics Approval Committee was obtained in advance. The collected blood samples were subjected to ultracentrifugation at approximately 1000 rpm for 5 min. The resulting supernatant blood plasma, obtained after centrifugation, was used for cortisol level determination using the DPV technique with the modified MIP@AgNPs sensor. Before analysis, the blood plasma samples were mixed with varying concentrations of cortisol at a pH of 7.4 to create spiked samples for calibration and validation purposes.

### 2.4 Preparation of Silver Nanoparticles

The synthesis of AgNPs was carried out using the widely recognized Turkevich sodium citrate method.^28^ Sodium citrate solution was added dropwise to AgNO_3_ (1.0 × 10^–3^ M) solution and heated to the boiling point. As a result of this reaction, the solution gradually changed color to a grayish-yellow hue, indicating the reduction of Ag^+^ ions. Continuation of the heating process for 15 min was followed by the cooling of the solution to room temperature. To determine the average silver core diameter (*D*) of each nanoparticle sample, transmission electron microscopy (TEM) images were captured. In order to calculate the average diameter, TEM images of at least 100 particles were taken and the particle size distribution was established. The standard deviation for each nanoparticle sample was determined by averaging the sizes of the particles obtained from the TEM images.

Concentration of AgNPs was calculated by usage of the TEM image data.^29^ Firstly, the number of Ag atoms per nanoparticle (N) was determined using eq 1. Then, eq 2 was employed to calculate the concentration of AgNPs (C). In the equations, ρ represents the density of Ag (fcc, 10.49 g/cm^3^), M denotes the atomic weight of Ag, D stands for the diameter of AgNPs, *N*_T_ refers to the total number of Ag atoms, V represents the volume of the solution, and N_A_ corresponds to Avogadro’s number. Hydrodynamic size of the AgNPs was estimated by conducting a dynamic light scattering (DLS) analysis by a Nano Zetasizer Instrument (NanoS, Malvern Instruments). The analysis was performed in triplicate (*n* = 3) to ensure accuracy and reliability.
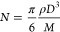
1
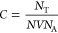
2

### 2.5 *N*-Methacryloyl-(l)-histidine Methyl Ester (MAH) Synthesis

In a brief summary, the experimental procedure involved the following steps: (1) dissolving 5.0 g of l-histidine methyl ester and 0.2 g of hydroquinone in a 100 mL solution of CH_2_Cl_2_; (2) cooling the solution to 0 °C; (3) adding 12.7 g of triethylamine to the cooled solution; (4) slowly pouring 5.0 mL of methacryloyl chloride into the solution under an inert atmosphere; (5) magnetic stirring of the mixture at a temperature of 25 °C for 2 h; (6) using a 10% NaOH solution to extract any unreacted methacryloyl chloride; (7) evaporating the aqueous phase using a rotary evaporator; and (8) finally, dissolving the resulting residue in ethanol.^30^

### 2.6 Polymer-Coated PGE Preparation

The preparation process for MIP@AgNPs, NIP@AgNPs, and MIP sensors on PGE 2.0/HB tips was summarized as follows: (1) cleaning the PGE surface: the PGE chip’s surface was cleaned by immersing it in 10 mL of pure ethyl alcohol and deionized water for 5 min, followed by drying at room temperature. (2) Preparation of AgNPs-containing cortisol-imprinted the MIP@AgNPs sensor: a nanofilm was formed on the surface of the PGE. Firstly, the MAH-AgNP prepolymerization complex was prepared, where AgNPs were coordinated with the functional monomer (0.1:1 mmol). The complex formation was measured using a UV–vis spectrophotometer. Then, the template cortisol (0.01 mmol) interacted with the MAH-AgNPs prepolymerization complex to form the MAH-AgNP-cortisol precomplex. The functional monomer MAH (1 mmol), crosslinker EGDMA (0.01 mmol), and hydrophilicity provider HEMA (0.4 mmol) were added, along with the AIBN initiator (2.5 mg). The tips were submerged in the monomer solution, and UV light (365 nm, 100 W) was used to initiate the polymerization process. The tips were left for 1 h to convert the monomer mixture into polymeric films. (3) Desorption of cortisol: to remove cortisol from the surface, the tips coated with the MIP@AgNPs sensor were washed in a desorption solution (methyl alcohol and acetic acid, 1:1 v/v) by shaking in a shaking incubator at 90 rpm and room temperature for 2 h. After the desorption process, the sensor surfaces were washed with a mixture of DI water and pure ethanol for 2 h and dried at 40 °C. (4) Preparation of the AgNP-free cortisol-imprinted MIP sensor (MIP sensor): the same polymerization technique as the one used for the MIP@AgNPs sensor was followed but without the use of AgNPs. Preparation of the nonimprinted NIP@AgNPs sensor: the same steps as those in the MIP@AgNPs sensor preparation were carried out but without the presence of cortisol molecules. This sensor served as a control for comparison and by following these procedures, MIP@AgNPs, NIP@AgNPs, and MIP sensors were successfully prepared on PGE tips for further analyses and experimentation.

### 2.7 Polymer-Coated PGE Characterization

The surface wettability of the bare PGE, MIP@AgNPs, NIP@AgNPs, and MIP sensors was measured by a Kruss DSA100 contact angle (CA) instrument (Hamburg, Germany) using the sessile drop method. Calculation of the average drop angles was performed using DSA2 software by measuring the CAs from the different parts of the sensor surfaces. The surface morphology of bare PGE, MIP@AgNPs, NIP@AgNPs, and MIP sensors was characterized by scanning electron microscopy (SEM; JSM-6400, JEOL) after coating with a thin gold-palladium (Au–Pd) alloy.

## 3 Results and Discussion

### 3.1 Characterization Studies

**Figure 2:**
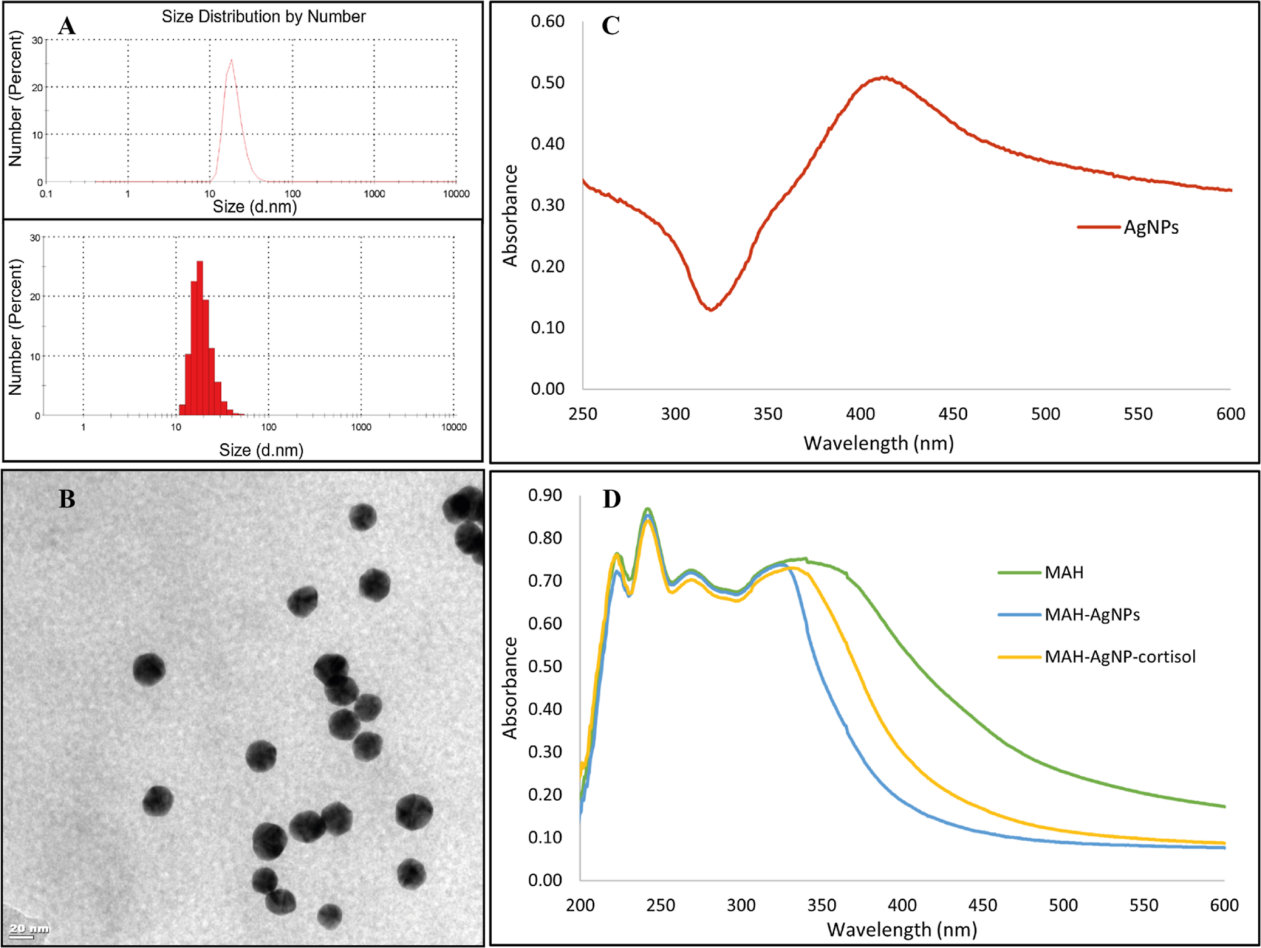
(A) Size distribution by the number of AgNPs, (B) TEM image of AgNPs, (C) UV–vis spectrum of AgNPs, (D) UV–vis spectra of the MAH monomer and the MAH-AgNP and MAH-AgNP-cortisol precomplexes.

SEM was used to study the surface morphology of the electrodes, and TEM was used to determine the size and shape of the AgNPs. The TEM image of the AgNPs sample was obtained using a 300 keV/FEG transmission electron microscope (FEI Tecnai-G2-F30). In Figure 2A, spheroidal AgNPs were determined with a homogeneous average size distribution (polydispersity index = 0.249), and no aggregation was observed due to the absence of particles of different sizes. The average size of AgNPs was determined as 39.45 ± 5.05 nm by measurements repeated three times (*n* = 3). A TEM image of the AgNPs approximately measured the concentration of AgNPs. The estimated diameter from the TEM measurements was 35.84 ± 1.29 nm. Figure 2B shows the TEM image of the AgNPs. The concentration of the AgNPs was calculated according to eq 2, mentioned previously. The concentration of the AgNPs solution was estimated to be 1.15 × 10^–7^ M. Characterization of the electrodes can provide information about the modified electrodes’ surface properties and electrocatalytic activity. The MAH monomer was mixed with AgNPs to form the MAH-AgNPs precomplex. MAH-AgNP and MAH-AgNPs-cortisol precomplexes were measured by a UV–vis spectrophotometer (Shimadzu UV-1601, Kyoto, Japan). As seen in Figure 2C, the band at 421 nm is recorded for AgNPs. The band of the MAH monomer recorded at 350 nm (Figure 2D) shifted to 344 nm after complexing with the MAH monomer, confirming the coordination with AgNPs. Similarly, after complexing with cortisol, shifting to the long wavelength was observed.

**Figure 3:**
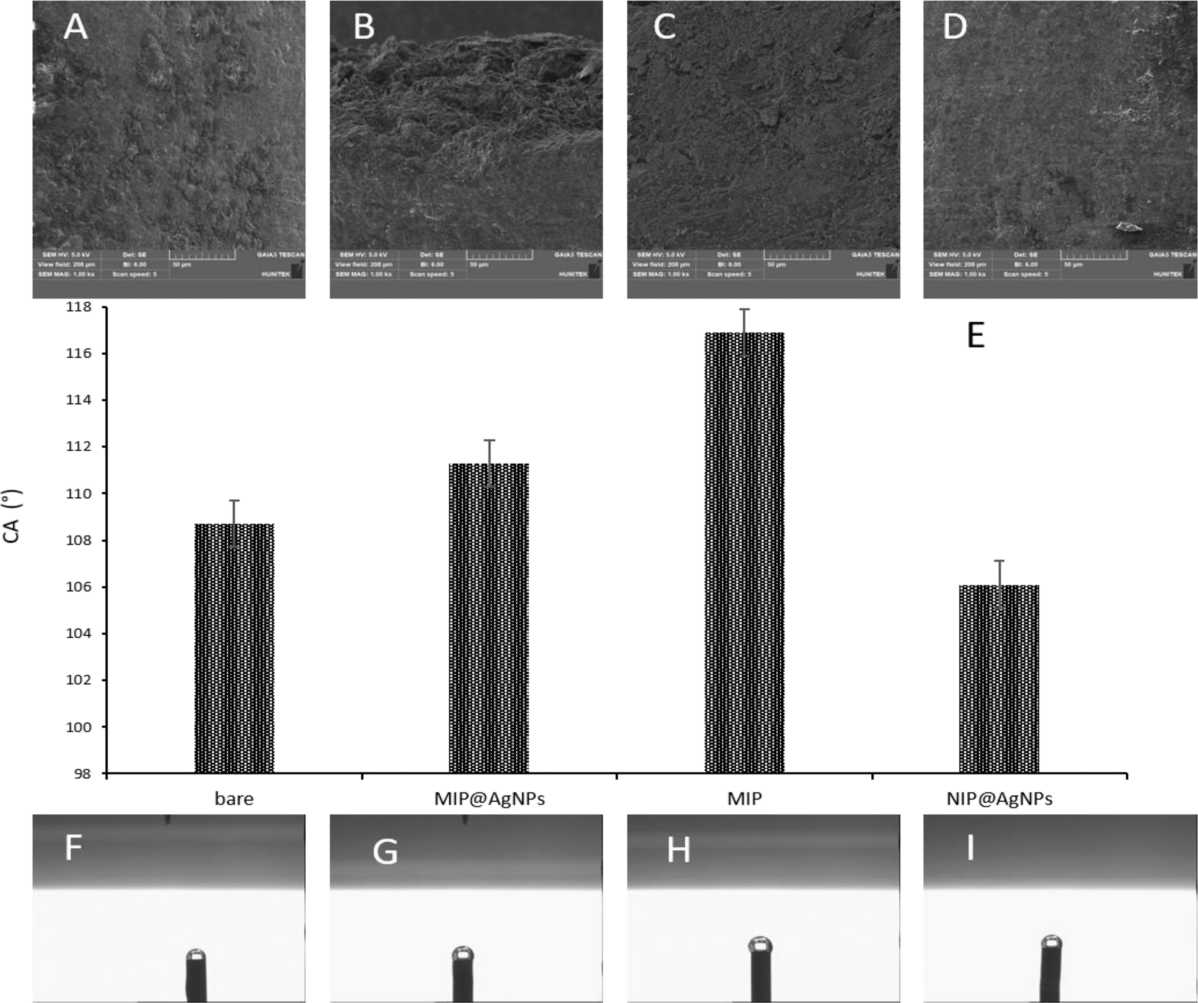
Surface characterization of PGEs. SEM images of PGEs: (A) bare PGE, (B) MIP@AgNPs sensor, (C) MIP sensor, (D) NIP@AgNPs sensor, and (E) column bar diagram of PGEs’ CA values. CA images of PGEs: (F) bare PGE, (G) MIP@AgNPs sensor, (H) MIP sensor, and (I) NIP@AgNPs sensor.

Figure 3 represents the surface characterization of PGEs. The SEM images at 5k× magnification values of PGEs are shown in Figure 3A–D, and the CA images of PGEs are shown in Figure 3F–I. The estimated CA values were calculated at 108.7° ± 1.04 for the bare PGE, 111.3° ± 1.25 for the MIP@AgNPs, 106.1° ± 0.92 for the NIP@AgNPs, and 116.9° ± 1.35 for the MIP sensor (Figure 3E). It was noticed that the hydrophobicity of the MIP@AgNPs sensor surface was increased compared to that of the NIP@AgNPs as a result of the cortisol coordination with the MAH monomer. Besides, it was observed that AgNPs provided surface hydrophilicity according to the decreased CA results of the MIP@AgNPs compared to those of the MIP sensor.

### 3.2 Kinetic Analysis and Optimization of the MIP@AgNPs Sensor

Due to the presence of cortisol in the brain and its electroactivity at a pH range of 7.2–7.4, electrochemical techniques indicate the highest activity around a pH of 7.4. A standard stock solution of cortisol with a concentration of 189.6 mg/mL was prepared using DI water and PBS at the same pH. This stock solution was stored at 4 °C in a refrigerator. Various concentrations of cortisol were then prepared from the stock solution for the calibration curve, ranging from 0.395 to 3.96 nM, through dilution with the buffer solution. The kinetic analysis was conducted using the MIP@AgNPs sensor with five different concentrations of cortisol (0.395, 0.791, 1.32, 2.64, and 3.96 nM) in 0.1 M PBS at a pH of 7.4. These concentrations were measured using the DPV technique with the starting potential and the stop potential set at −0.6 and +0.6 V, respectively, and a modulation time of 0.05 s. To enhance the electrode affinity, the cortisol molecules were desorbed from the MIP@AgNPs sensor cavities between each measurement using a desorption solution consisting of methanol and acetic acid in a ratio of 9:1 v/v. The measurements were carried out three times in order to compute the relative standard deviation (RSD %) and evaluate repeatability accuracy.

### 3.3 MIP@AgNPs Sensor Electrochemical Response

**Figure 4:**
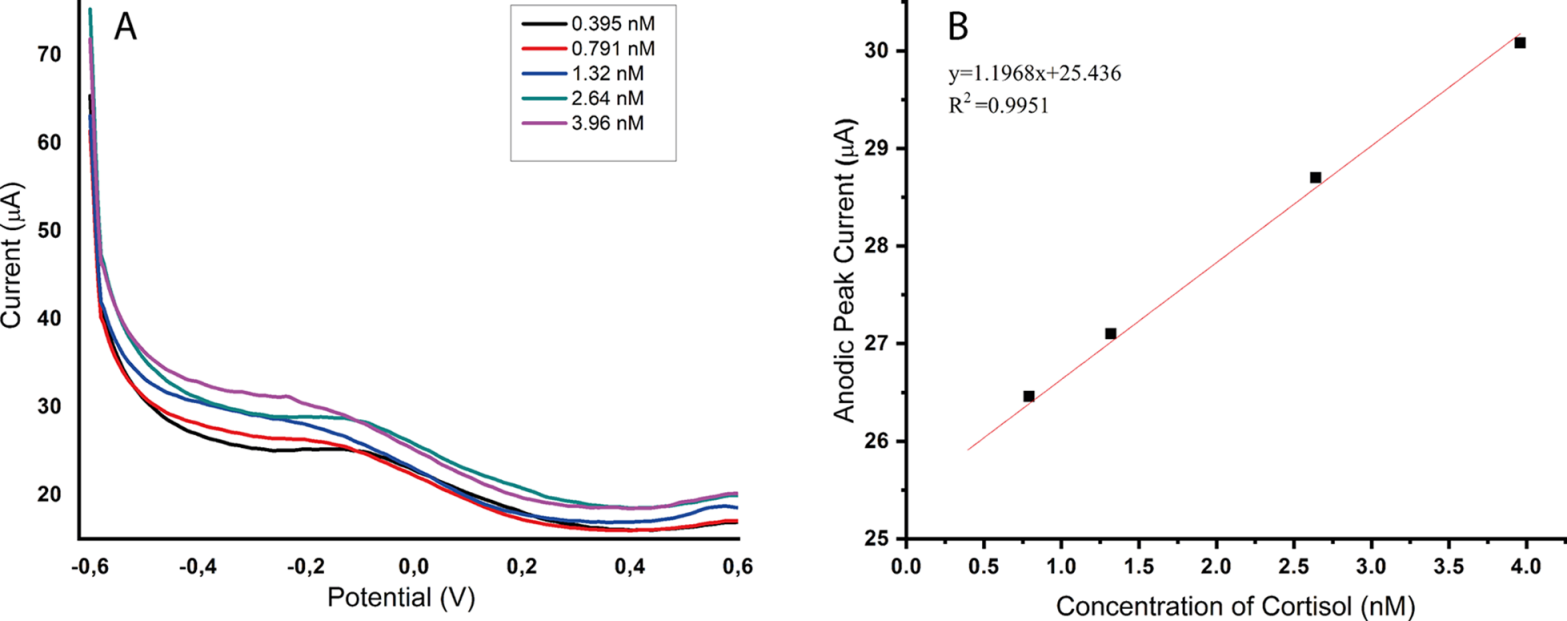
Cortisol detection and kinetic parameters. (A) MIP@AgNPs sensor response for different cortisol concentrations by DPV and (B) calibration curve of different cortisol concentrations (0.395–3.96 nM).

The negatively charged modified polymer electrode surface exhibited a favorable interaction with the positively charged cortisol molecules, resulting in enhanced electrode selectivity toward the analyte. This electrostatic attraction facilitated the adsorption of cortisol molecules onto the electrode surface, contributing improved specificity of the electrode for detecting cortisol. Since MAH contains the functional group histidine, it was used as a complexing agent for both the AgNPs and the cortisol molecule in one mode. Figure 4 shows the voltammograms of five different cortisol concentrations (0.395, 0.791, 1.32, 2.64, and 3.96 nM) obtained by DPV, a highly sensitive technique among other analytical techniques. The peak potential was observed around −0.15 V, where the increase in cortisol concentration enhanced its oxidation. This relationship between the cortisol concentration and the oxidation signal allows for quantitative determination of cortisol levels. Their results showed that the MIP@AgNPs sensor was suitable for practical and fast diagnostics of cortisol levels without sample pretreatment using only a small sample volume.

With a regression coefficient of 0.9951 (Table 1) suggesting a strong linear relationship between the cortisol concentration and the response signal, the calibration curve (Figure 4) for the voltammetric detection of cortisol seems to be of good quality. The slope of the curve (4.695 × 10^–6^) is within the acceptable range for an electrochemical sensor and denotes a cortisol-detection sensitivity. The approach is able to detect and precisely quantify cortisol at low concentrations, which is superior to other studies,^30^ as shown by the LOD and LOQ values of 0.214 and 0.641 nM, respectively. Also, the measurements’ standard deviation of 1.092 × 10^–7^ indicates acceptable accuracy and reproducibility. These results show that the MAH monomer can enhance the electrode selectivity for cortisol molecules. Overall, these findings imply that the developed cortisol voltammetric detection method in this study is accurate and suited for measuring cortisol concentrations with high sensitivity.

**Table 1:** Figures of Merit of Cortisol Determination by the DPV Technique

technique	*R*^2^	*m* (μA/nM)	SD (μA)	LR^a^ (nM)	LOD (nM)	LOQ (nM)
DPV	0.9951	4.695 × 10^–6^	1.092 × 10^–7^	0.395–3.96	0.214	0.641

a Linear range.

### 3.4 Selectivity and Imprinting Efficiency Assessment

In recent years, sensors have attracted great interest as biological recognition elements because of their integration with molecularly imprinted nanoparticles. Molecular imprinting is used to create recognition sites for selective recognition in a macromolecular matrix using a template molecule.^31^ MIPs have stable chemical and physical structures and have many superior properties such as advanced mechanical properties, resistance to high temperature and high pressure, strong resistance to acids and alkalis, easy synthesis, long-term performance life, reusability, and recycling. The polymerization process is carried out around the precomplexes containing the template molecule by adding the initiator and crosslinker. The template is then removed to create specific three-dimensional shapes. The template can interact with the imprinted sites multiple times without losing performance. These advantages make MIPs a promising platform for developing highly efficient and durable sensor systems.

**Figure 5:**
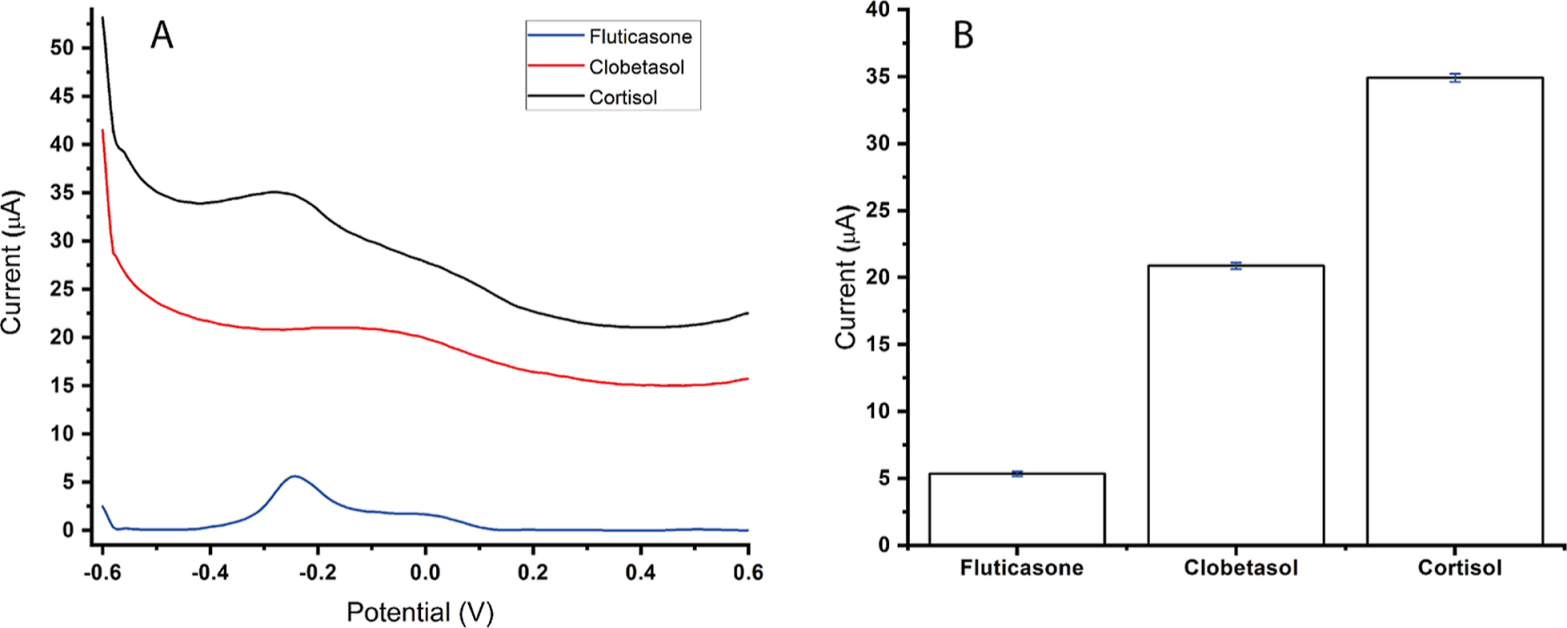
Selectivity study. (A) MIP@AgNPs sensor response for cortisol (2.64 nM), fluticasone (2.64 nM), and clobetasol (2.64 nM) competitors and (B) error bar for MIP@AgNPs sensor response (*n* = 3).

The selectivity assessment of the MIP@AgNPs sensor involved the detection of other neurotransmitters, specifically fluticasone and clobetasol propionate, which exhibit similar chemical and structural behavior to cortisol. To evaluate the performance of the MIP@AgNPs sensor, solutions containing 2.64 nM of cortisol, fluticasone, and clobetasol propionate were prepared in 0.1 M PBS at a pH of 7.4. The same procedure and kinetic study technique which were used earlier were applied to analyze the competing behavior of these molecules. To observe the imprinting effect and evaluate selectivity, the MIP@AgNPs sensor was exposed to a neurotransmitter concentration of 2.64 nM. Additionally, competitive adsorption studies were conducted with competitor molecules, namely, fluticasone and clobetasol propionate, both at a concentration of 2.64 nM. The imprinting efficiency estimation was conducted by comparing voltammograms of the MIP@AgNPs sensor to that of the NIP@AgNPs sensor in detecting cortisol at a concentration of 2.64 nM. Figure 5 illustrates the results of this comparison, providing insights into the selectivity and imprinting efficiency of the MIP@AgNPs sensor for cortisol detection.

Given the low concentration of cortisol in human plasma and the presence of numerous interfering compounds in the matrix, there is a critical need for a selective measurement technique. A cost-effective, simple, and efficient method for specifically detecting cortisol is imperative. The MIP@AgNPs sensor developed using the MAH monomer, which incorporates histidine, offers the ability to distinguish cortisol from other neurotransmitter molecules, even at nanomolar concentrations. This selectivity is crucial in ensuring accurate and reliable cortisol detection despite the complex and interfering nature of the sample matrix.

**Figure 6:**
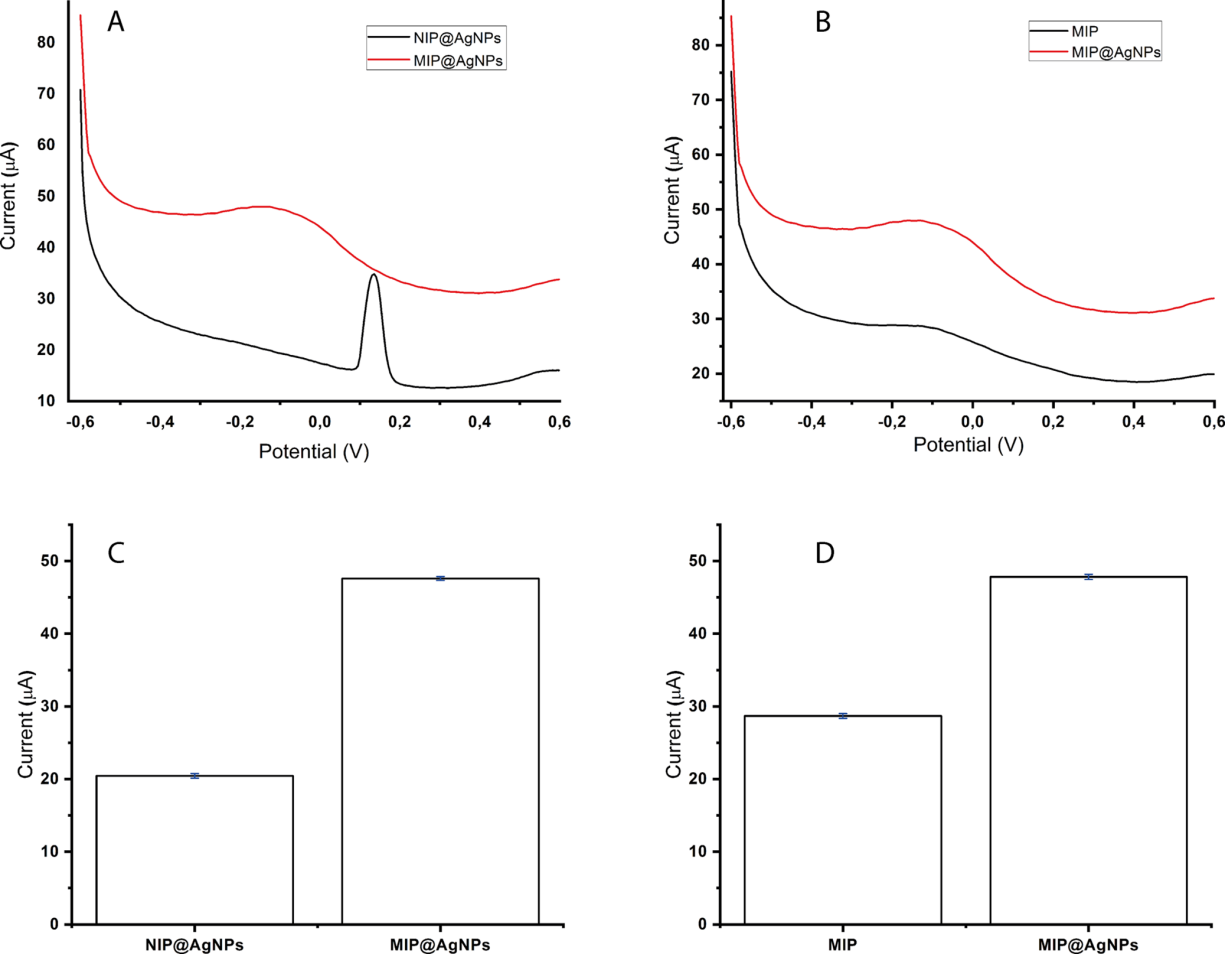
Modified electrode response, (A) MIP@AgNPs and NIP@AgNPs sensors’ response for 2.64 nM cortisol, (B) error bar for MIP@AgNPs and MIP sensors’ response (*n* = 3), (C) MIP@AgNPs and NIP@AgNPs sensors’ response for 2.64 nM cortisol, and (D) error bar for MIP and MIP@AgNPs sensors’ response (*n* = 3).

The MIP electrode serves as a differentiation element, creating special cavities that precisely match the template molecule in terms of shape and size. These cavities play a crucial role in imparting selectivity and improving the efficiency of the analyte-binding process. Figure 6 demonstrates the evaluation of cortisol detection using the NIP@AgNPs, MIP@AgNPs, and MIP electrodes. The study highlights the electrodes’ high sensitivity, as is evident from their cortisol response. To assess the imprinting efficiency, the three electrode sensors were tested with 2.64 nM cortisol. The comparison between the MIP@AgNPs sensor and the NIP@AgNPs sensor for detecting cortisol (2.64 nM) is depicted in Figure 6A. Similarly, the efficiency of the MIP@AgNPs sensor in detecting cortisol (2.64 nM) was compared to that of the MIP sensor, as shown in Figure 6B. These comparisons provide insights into the superior performance and selectivity of the MIP@AgNPs sensor for cortisol detection.

### 3.5 MIP PGE Sensor Repeatability Study

**Figure 7:**
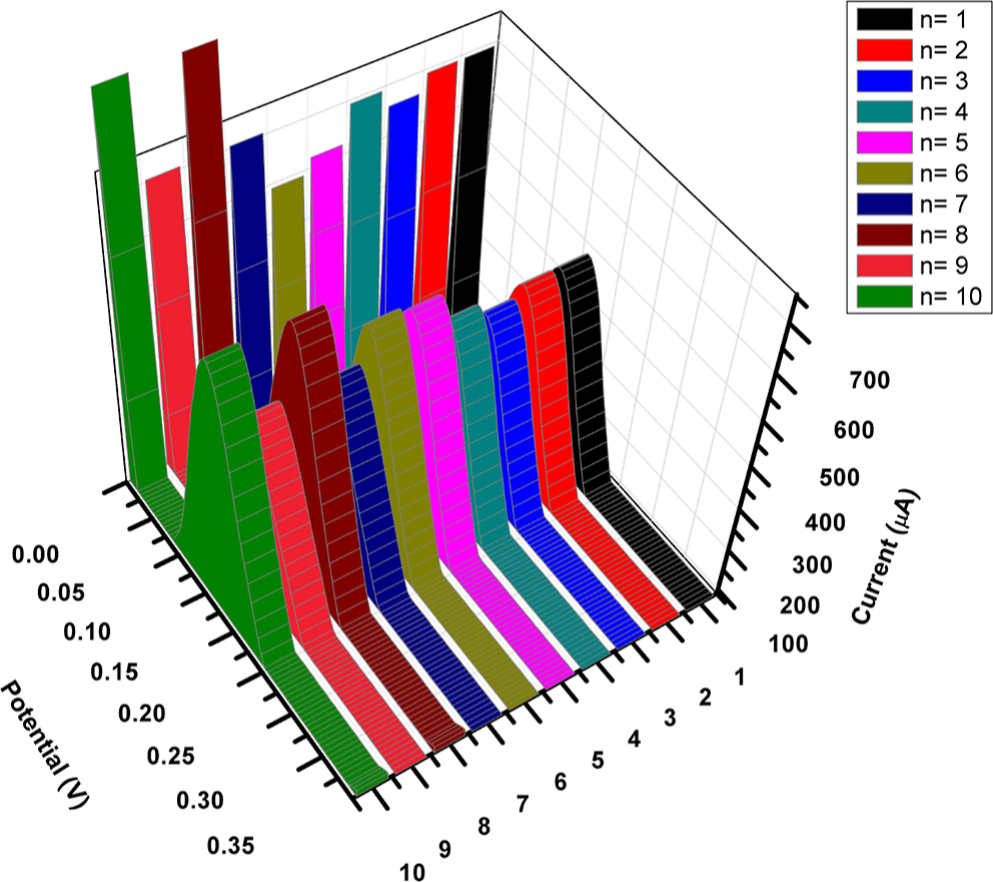
MIP@AgNPs sensor repeatability study. Response of the sensor for 2.64 nM cortisol concentration. (*n* = 10).

The fouling of the imprinted electrode surface during the measurements or regeneration steps could limit the detection of the analyte by the polymer-modified electrode surface. As a result, the imprinted cavities formed for the cortisol on the PGE surface may become passivated. This fouling and passivation can impede the effective functioning of the modified electrode and hinder accurate analyte detection. Therefore, it is essential to address and overcome these challenges in order to maintain the performance and functionality of the modified electrode for successful and reliable analysis. In order to evaluate the repeatability of the MIP@AgNPs sensor, a test can be performed where the electrode is used to measure cortisol concentrations multiple times. The test should be performed with the same cortisol concentration and under the same conditions to minimize variations. The electrode capacity for the number of measurements for each electrode can be determined by measuring the cortisol concentration at a specific interval, for example, every hour, over a certain period of time, for example, 24 h, and comparing the results. If the results are consistent and reproducible, it can be concluded that the electrode has good repeatability. Additionally, the coefficient of variation can be used to express the repeatability of the electrode. After taking the standard deviation of the measurements, the coefficient of variation was calculated by dividing by the mean of the measurements and then multiplying by 100%.^32^ The repeatability of the MIP@AgNPs sensor was tested by utilizing a 2.64 nM cortisol solution, and the MIP@AgNPs sensor response was plotted as potential versus current applied, as seen in Figure 7, and a 2.5% coefficient of variation was obtained for the MIP@AgNPs sensor. A coefficient of variation value of less than 5% is generally considered acceptable for most analytical measurements as it indicates a high degree of reproducibility and precision.^33^ The excellent reusability and reproducibility of the procedure indicated by an RSD % result of less than 1.5 for the 10 analyses performed consecutively confirm the three-dimensional stability of the MIP@AgNPs sensor. This consistent performance over multiple analyses showcases its potential for robust and reliable cortisol detection in various practical applications.

MIP@AgNPs sensor stability was assessed for cortisol detection by immersing the PGE in a cortisol solution with a concentration of 2.64 nM. The detection was performed using the DPV method for a total of 10 measurements (*n* = 10). Between each measurement, the electrode underwent a desorption step by immersion in a desorption solution consisting of methyl alcohol and acetic acid in a ratio of 1:9 (v/v). Obtained voltammograms from the ten measurements were evaluated using the equation below.
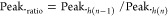
3In eq 3, peak height is referred to as “*h*” and “*n*” refers to the number of carried measurements. The experimental outcomes indicate that a relative standard deviation value of 1.27% for cortisol, obtained for the current (*I*) signal, implies high electrode stability. The sensor response was obtained 10 times for the same cortisol concentration of the MIP@AgNPs sensor with high efficiency (98%). That means that the MIP@AgNPs sensor can be reused 10 times without loss of efficiency and affinity.

### 3.6 Cortisol Detection in Human Plasma

**Figure 8:**
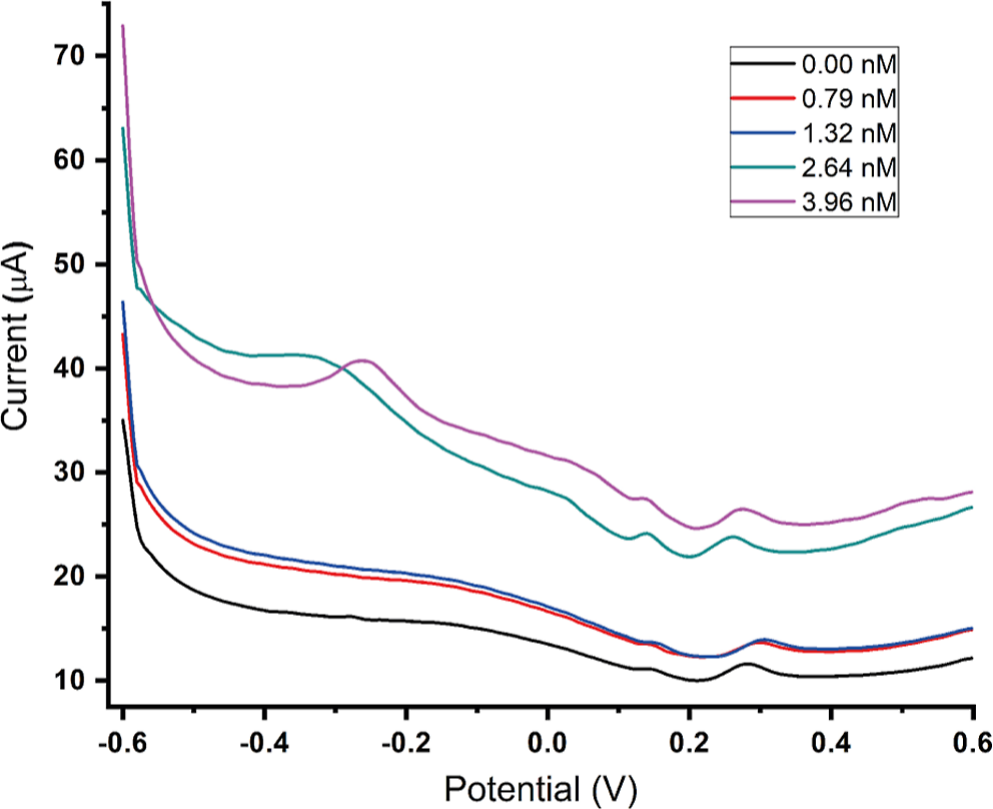
Spiked plasma sample response ranges from 0.395 to 3.96 nM cortisol.

The MIP@AgNPs synthesized sensor was specifically designed to recognize cortisol molecules in human plasma samples, distinguishing them from other molecules with similar functional groups, such as steroids. This sensor offers high selectivity and sensitivity for the detection of cortisol. Its unique design and composition allow for precise and accurate identification of cortisol, even in complex biological samples like human plasma. The voltammetric method was used to quantify the cortisol molecules in human blood plasma, where cortisol concentration levels range from 5 to 25 micrograms per deciliter (μg/dL) or from 140 to 690 nanomoles per liter (nmol/L) in the morning and from 3 to 15 μg/dL or from 83 to 417 nmol/L at night.^34^ The MIP@AgNPs sensor was preserved with human blood plasma solutions spiked with different concentrations of cortisol solutions (0.395, 0.791, 1.32, 2.64, and 3.96 nM) prepared to measure the cortisol concentration level in PBS at a pH of 7.4. Figure 8 shows a slight shift in the nonspiked sample due to the natural occurrence of fluticasone and clobetasol propionate molecules in the serum plasma sample. The reason for the change in peak height (current response) is the increase in cortisol concentrations spiked into the plasma samples. . The lowest peak height was reported when the plasma sample without cortisol solution was analyzed. Therefore, it has been determined that the generated cortisol-imprinted MIP@AgNPs sensor modified with silver nanoparticles is highly selective, rapidly responsive, simple to use, reusable, and sensitive for cortisol detection in a human plasma solution. An amino acid-containing MIP@AgNPs sensor that was manufactured without complicated coupling methods and labeling processes was used to detect cortisol molecules. These characteristics make the sensor an attractive and practical tool for accurate and efficient measurement of cortisol levels.

The objective of this study is to develop and enhance a MIP@AgNPs sensor for the detection of cortisol in both aqueous solutions and human plasma samples. By utilizing MAH on the electrode surface, the selectivity of the sensor is significantly improved, allowing for the detection of cortisol even in ultratrace amounts within the samples. MIPs provide highly desirable functional chemical groups with imprinted sites that contribute to the selective recognition of template molecules. In comparison to recognition materials like antibodies and enzymes, the synthesis of MIPs offers several advantages, including ease of synthesis, lower cost, and reduced time requirements. These advantages overcome many of the known disadvantages associated with alternative recognition materials. The high recognition ability of the MIP@AgNPs sensor for cortisol molecules, even within complex matrices such as human plasma, is of significant importance for various biological studies. The sensor’s ability to accurately detect cortisol in complicated samples opens up opportunities for investigating cortisol’s role in biological process applications.

The LOD is a measure of the sensitivity of a detection method, and a lower LOD indicates a higher level of sensitivity. The detection of cortisol using the MIP@AgNPs sensor has yielded the best LOD compared to other research articles in Table 2. For example, one study that used gold based on PGEs reported a LOD of 27.6 nM for cortisol detection using an electrochemical method, while our research achieved a LOD of 0.214 nM using the MIP@AgNPs sensor. This indicates that our method is twenty times more sensitive. Another study used a different type of electrochemical sensor electrode (carbon-imprinted polymer) for cortisol detection and reported a LOD of 9997.2 nM using the same detection technique, DPV.

**Table 2:** MIP Electrode Sensors for Cortisol Detection

method	electrode	LOD (nM)	reference
CV	antibody immobilized on Au-PGE	27.6	(35)
DPV	AuNPs RGo C	138	(36)
CV	carbon-imprinted polymer	9997.2	(37)
DPV	AuNPs/MoS_2_/AuNPs	110.4	(38)
DPV	graphene Au NPs	27.5	(39)
SWV	Au nanowires	204	(40)
DPV	MIP@AgNPs PGE sensor	0.214	this study

In comparison to other research articles, our study demonstrates a significantly lower LOD for cortisol detection using the MIP@AgNPs sensor. Furthermore, our research also showed high selectivity and reproducibility for cortisol detection using the MIP@AgNPs sensor. This indicates that the method is reliable and specific to detecting cortisol, which is important in clinical applications where specificity and reproducibility are crucial. Overall, our research on cortisol detection using the MIP@AgNPs sensor has the best LOD among the research articles mentioned in the table. This method offers a high sensitivity, selectivity, and reproducibility, making it a promising approach for cortisol detection in various clinical applications. In addition, cortisol detection in the presence of similar molecules such as fluticasone and clobetasol propionate was achieved by the MIP@AgNPs sensor with excellent selectivity and sensitivity.

## 4 Conclusions

In conclusion, the detection of cortisol using molecularly imprinted poly(HEMA-MAH)-coated pencil graphite electrodes modified with AgNPs was successfully achieved with a low detection limit. Additionally, selectivity was also demonstrated by detecting the competitors of cortisol. The method was also applied to detect cortisol in human plasma. Overall, the results indicate that this method is a promising sensitive and selective cortisol detection approach. We produced an electrochemical MIP sensor with AgNPs for the specific detection of cortisol molecules due to creation of imprinted cavities on the electrode surfaces, resulting in a structure with binding sites that are highly specific to the template molecule. This specificity allows for detecting cortisol at low concentrations, making the MIP@AgNPs a valuable sensor in biomedical research and clinical diagnostics applications. Additionally, the MIP@AgNPs sensor is relatively simple and inexpensive to produce, making them a cost-effective option for detecting cortisol. Furthermore, the selectivity of the synthesized electrode was also measured with increased sensitivity and selectivity for the cortisol molecule detection even in the presence of fluticasone and clobetasol propionate molecules due to the polymer film on the PGEs. It has been successfully used to detect cortisol in human blood plasma samples. When compared to conventional electrode sensors for cortisol detection, the polymer film that is formed on the electrode surface offers advantages while lowering the detection of fluticasone and clobetasol molecules, which are utilized as interferences. Furthermore, modifying these PGEs by adding AgNPs due to their high surface area also improved the sensitivity and selectivity for cortisol analysis. The detection of cortisol molecules with the presence of the cortisol competitors such as fluticasone and clobetasol was achieved in a value less than 0.22 nM. The detection was achieved in a short period of time with high-sensitivity and low-detection limits of 0.214 nM by the MIP@AgNPs sensor without using any extra processes such as spacer arms for the immobilization of the ligands. The results indicate that the MIP@AgNPs sensor has the best performance in terms of sensitivity and selectivity among all three types of electrodes. This finding highlights the efficacy of incorporating AgNPs into the MIP structure for cortisol detection. In conclusion, a cortisol-imprinted electrochemical sensor modified with AgNPs was manufactured, and real-time and sensitive cortisol determination were conducted both from an aqueous solution and in a multifaceted environment, i.e., human plasma without the need of extra complex procedures like ligand immobilization. The developed method has great potential for detecting sensitive and selective cortisol in biological samples.
